# Changes in optical coherence tomography biomarkers in eyes with advanced idiopathic epiretinal membrane treated with dexamethasone implantation

**DOI:** 10.1186/s12886-024-03312-0

**Published:** 2024-02-02

**Authors:** Zetong Nie, Wenbo Li, Naxin Duan, Jiaoting Wei, Xiang Zhang, Haoxin Guo, Siqiong Bai, Xiaorong Li, Bojie Hu

**Affiliations:** Tianjin Key Laboratory of Retinal Functions and Diseases, Tianjin Branch of National Clinical Research Center for Ocular Disease, Eye Institute and School of Optometry, Tianjin Medical University Eye Hospital, No. 251, Fukang Road, Nankai District, Tianjin, 300384 China

**Keywords:** Idiopathic epiretinal membrane, Dexamethasone, Vitrectomy, Optical coherence tomography biomarker, Central macular thickness, Disorganization of the retinal inner layers

## Abstract

**Purpose:**

To investigate the effects of vitrectomy and intravitreal dexamethasone (DEX) implantation on retinal biomarkers in patients with advanced idiopathic epiretinal membrane (IERM) and to evaluate this treatment’s anatomical and functional outcomes.

**Methods:**

This retrospective study included 41 patients with advanced IERM who underwent vitrectomy and were divided into a pars plana vitrectomy (PPV) group (20 eyes) and a dexamethasone (DEX) group (21 eyes) based on intravitreal DEX implantation. We collected data on best-corrected visual acuity (BCVA), central macular thickness (CMT), disorganization of the retinal inner layers (DRIL), subretinal fluid, intraretinal cystoid changes (IRC), integrity of the inner-outer segment layer, and intraocular pressure.

**Results:**

BCVA improved significantly in both groups; the DEX group had a higher visual acuity gain at 1 and 6 months (*P* = 0.002 and 0.023, respectively). Postoperative CMT gradually decreased in both groups, with the DEX group showing a greater decrease at 1 and 6 months (*P* = 0.009 and 0.033, respectively). Six months after surgery, the DRIL and IRC grades in the DEX group were significantly improved compared to those in the PPV group (*P* = 0.037 and 0.038, respectively). Multivariate regression analyses revealed that patients with intraoperative DEX implants were more likely to have a significant CMT reduction (≥ 100 μm) from baseline (odds ratio (OR), 9.44; 95% confidence intervals (CI), 1.58–56.56; *P* = 0.014) at 6 months and less likely to exhibit DRIL at 6 months postoperatively (OR, 0.08; 95% CI, 0.01–0.68; *P* = 0.021).

**Conclusion:**

Vitrectomy combined with intravitreal DEX implantation facilitates the recovery of postoperative visual acuity and improvement of anatomical outcomes in patients with advanced IERM, effectively reducing CMT and improving DRIL.

## Background

Epiretinal membrane (ERM) is a prevalent vitreoretinal disease typically affecting people aged 50 years and above. It is characterized by abnormal tissue proliferation on the retina’s inner surface, often resulting in visual impairment [1, 2]. The current mainstay of treatment is vitrectomy combined with epiretinal membrane peeling, which aims to remove the membrane and relieve retinal traction [3]. Govetto et al. (2017) classified idiopathic epiretinal membrane (IERM) into four stages based on the extent of macular foveal disruption observed on spectral domain optical coherence tomography (SD-OCT) imaging: stage 3 ERM is characterized by the absence of a foveal pit and the presence of continuous ectopic internal foveal layer (EIFL) with visible retinal layers; stage 4 ERM is characterized by the absence of a foveal pit and presence of continuous EIFL with disorganization of the retinal inner layers (DRIL) [4].

Advanced IERM was found to be more complex than the early stage, with patients at stage 3–4 IERM experiencing less visual and anatomical improvement, particularly those at stage 4, with significant retinal thickening, marked macular anatomical disruption, and complete foveal disorganization, including EIFL extension from the retina’s inner nuclear layer and the inner plexiform layer throughout the entire foveal region [5]. Iuliano et al. suggested that this state of retinal structural disruption observed in patients with advanced-stage IERM could predispose the tissue to further changes when exposed to mechanical stresses like epiretinal membrane peeling [6].

Numerous studies have demonstrated that anatomical changes, such as EIFL, macular edema, intraretinal cystoid changes (IRC), and DRIL, can affect postoperative visual acuity in patients with advanced IERM [7–13]. Several studies have reported positive anatomical and functional outcomes with dexamethasone (DEX) implantation in patients with ERM [14–17]. However, limited information is available concerning the anatomical changes in patients with advanced IERM undergoing DEX treatment. This study aimed to assess alterations in OCT biomarkers among patients with advanced IERM following vitrectomy combined with intravitreal DEX implantation and investigate whether DEX can enhance anatomical and functional prognosis in patients with advanced IERM.

## Methods

### Participants

This was a retrospective study conducted at the Tianjin Medical University Eye Hospital. In this study, we reviewed 265 patients who underwent vitrectomy combined with cataract ultrasound emulsification and intraocular lens (IOL) implantation surgery at Tianjin Medical University Eye Hospital between 2018 and 2022. According to Govetto et al., IERM is divided into four stages, of which stages 3 and 4 are considered advanced IERM. In this study, 41 patients with advanced IERM were included and divided into a pars plana vitrectomy (PPV) group (20 eyes) and a DEX group (21 eyes) according to whether they received intraoperative DEX implantation. We excluded 112 cases of early-stage IERM, 42 cases with incomplete medical records, 32 cases of retinal vein occlusion, 20 cases of diabetic retinopathy, 10 cases of lamellar macular hole 3 cases of glaucoma, 5 cases of retinal hole.

The inclusion criteria were: (1) reduction in best-corrected visual acuity (BCVA) with or without visual distortion, (2) presence of stage 3–4 IERM, as identified by SD-OCT, (3) lens opacity that impacted fundus visualization and required combined cataract surgery, and (4) agreement to participate in the research protocol. The exclusion criteria were: (1) secondary ERM diagnosis, (2) presence of other ocular diseases that may affect visual function, (3) history of intraocular surgery, and (4) OCT image quality affected by dioptric media opacity.

The preoperative data included age, sex, laterality, systemic history, symptom duration, slit-lamp microscopy, lens status, fundus examination, intraocular pressure (IOP), BCVA (logMAR), IERM stage and OCT. The OCT measurements included central macular thickness (CMT), DRIL grades [13], subretinal fluid (SRF), IRC grades [18], and integrity of the inner-outer segment layer (IS-OS). Postoperative data included BCVA, IOP, CMT, DRIL, SRF, IRC, and IS-OS integrity at 1 and 6 months after surgery.

SD-OCT (Heidelberg Engineering GmbH, Germany) was employed during each subsequent follow-up appointment. Three experienced ophthalmologists took part in the assessment of the OCT images: two attending physicians and one chief physician, all of whom underwent training together. The OCT images extracted from linear scans were assessed by the attending physicians who had no knowledge of the patient’s treatment approach, with any differences resolved by the chief physician.

### Surgical procedure

All procedures were performed using a standard three-port, 25-gauge PPV Constellation Vitrectomy System (Alcon, Fort Worth, TX, USA) by the same experienced surgeon. All the patients underwent cataract ultrasound emulsification with IOL implantation. Core vitrectomy was first performed, and posterior vitreous detachment was induced followed by triamcinolone acetonide staining. The macular epiretinal membrane was peeled off after 10 s of indocyanine green-assisted staining. At the end of the surgery, 0.7 mg of DEX was intravitreally implanted into the vitreous cavity in the DEX group.

### Statistical analysis

An independent sample t-test was used to compare the normally distributed quantitative data, whereas the Mann–Whitney U test was applied to abnormally distributed data. The chi-square and Fisher’s exact tests were applied to categorical data, whereas the rank-sum test was applied to rank data. Spearman correlation analysis and univariate logistic regression analysis were conducted to assess factors associated with the results. The variables with *P* < 0.05 in the univariate model were included for multivariate regression analysis. Statistical significance was set at *P* < 0.05. SPSS (version 25.0; IBM Corporation, Armonk, NY, USA) was used for all the statistical analyses.

## Results

### Patient characteristics

In this study, we included 41 eyes of 41 patients who had vitrectomy combined with ultrasound emulsification, IOL implantation, and ERM peeling. The PPV and DEX groups included 20 and 21 eyes, respectively. Demographic characteristics such as sex, age, laterality, symptom duration, and systemic history, as well as clinical characteristics including BCVA, IOP, and OCT data including ERM stage, CMT, DRIL, SRF, and IRC, were all comparable between the PPV and DEX group, with no significant differences observed (*P* > 0.05; Table 1).

**Table 1:** Comparison of baseline features between the two groups

	**PPV, *n* = 20**	**DEX, *n* = 21**	***T/Z***	***P***
**Gender (male/female)**	**6/14**	**6/15**	**-**	**0.920**
**Age (years)**	**68.35 ± 6.42**	**69.43 ± 6.03**	**0.56**	**0.582**
**Laterality(right/left)**	**9/11**	**7/14**	**-**	**0.530**
**Duration of symptoms (years)**	**1.00(0.50,2.00)**	**2.00(1.00,3.00)**	**1.38**	**0.169**
**hypertension (yes/no)**	**6/14**	**9/12**	**-**	**0.520**
**diabetes(yes/no)**	**3/17**	**1/20**	**-**	**0.343**
**BCVA(logMAR)**	**0.40(0.30,0.58)**	**0.70(0.40,0.78)**	**1.83**	**0.067**
**IOP (mmHg)**	**15.23 ± 3.12**	**14.40 ± 2.47**	**0.94**	**0.352**
**IERM stage(3/4)**	**4/16**	**5/16**	**-**	**1.000**
**CMT(μm)**	**461.40 ± 81.95**	**513.48 ± 117.47**	**1.64**	**0.109**
**DRIL(absent/mild/severe)**	**4/10/6**	**5/8/8**	**0.22**	**0.823**
**SRF(yes/no)**	**1/19**	**1/20**	**-**	**1.000**
**IRC(absent/mild/modrent/severe)**	**13/5/2/0**	**12/6/3/0**	**0.29**	**0.588**
**IS-OS disruption(yes/no)**	**9/11**	**5/16**	**-**	**0.197**

### The effect of dexamethasone on visual acuity

There were no significant differences in BCVA between the PPV and DEX groups preoperatively and at 1 and 6 months postoperatively. At 1 and 6 months postoperatively, the ΔBCVA (preoperative BCVA minus postoperative BCVA) was significantly higher in the DEX group than in the PPV group, and the differences were statistically significant (*P* = 0.002 and 0.023, respectively). And there were no statistically significant differences in the IOP between the two groups preoperatively and at 1 and 6 months postoperatively (Table 2).

**Table 2:** Comparison of 6-month visual acuity between the two groups

	**PPV, *n* = 20**	**DEX, *n* = 21**	***T/Z***	***P***
**BCVA(logMAR)**	**0.30(0.11,0.49)**	**0.22(0.10,0.40)**	**0.39**	**0.693**
**ΔBCVA**	**0.18(0.00,0.25)**	**0.30(0.19,0.58)**	**2.26**	0.023^*^
**IOP (mmHg)**	**14.33 ± 2.82**	**13.06 ± 2.87**	**1.43**	**0.162**

^*^P < 0.05

### The changes in OCT biomarkers with dexamethasone

There were no significant differences in CMT between the PPV and DEX groups preoperatively and at 1 and 6 months postoperatively. At 1 and 6 months postoperatively, ΔCMT (preoperative CMT minus postoperative CMT) was significantly higher in the DEX group than in the PPV group, and the differences were statistically significant (*P* = 0.009 and 0.033, respectively). After observing the OCT parameters, we found no significant differences in SRF and IS-OS disruption between the PPV and DEX groups preoperatively and postoperatively. At 6 months, DRIL was still present in 11 (55%) patients in the PPV group and 5 (23.8%) in the DEX group (*P* = 0.041). The grade of DRIL was significantly improved in the DEX group compared to that in the PPV group at 6 months, with a statistically significant difference (*P* = 0.037).

**Fig. 1:**
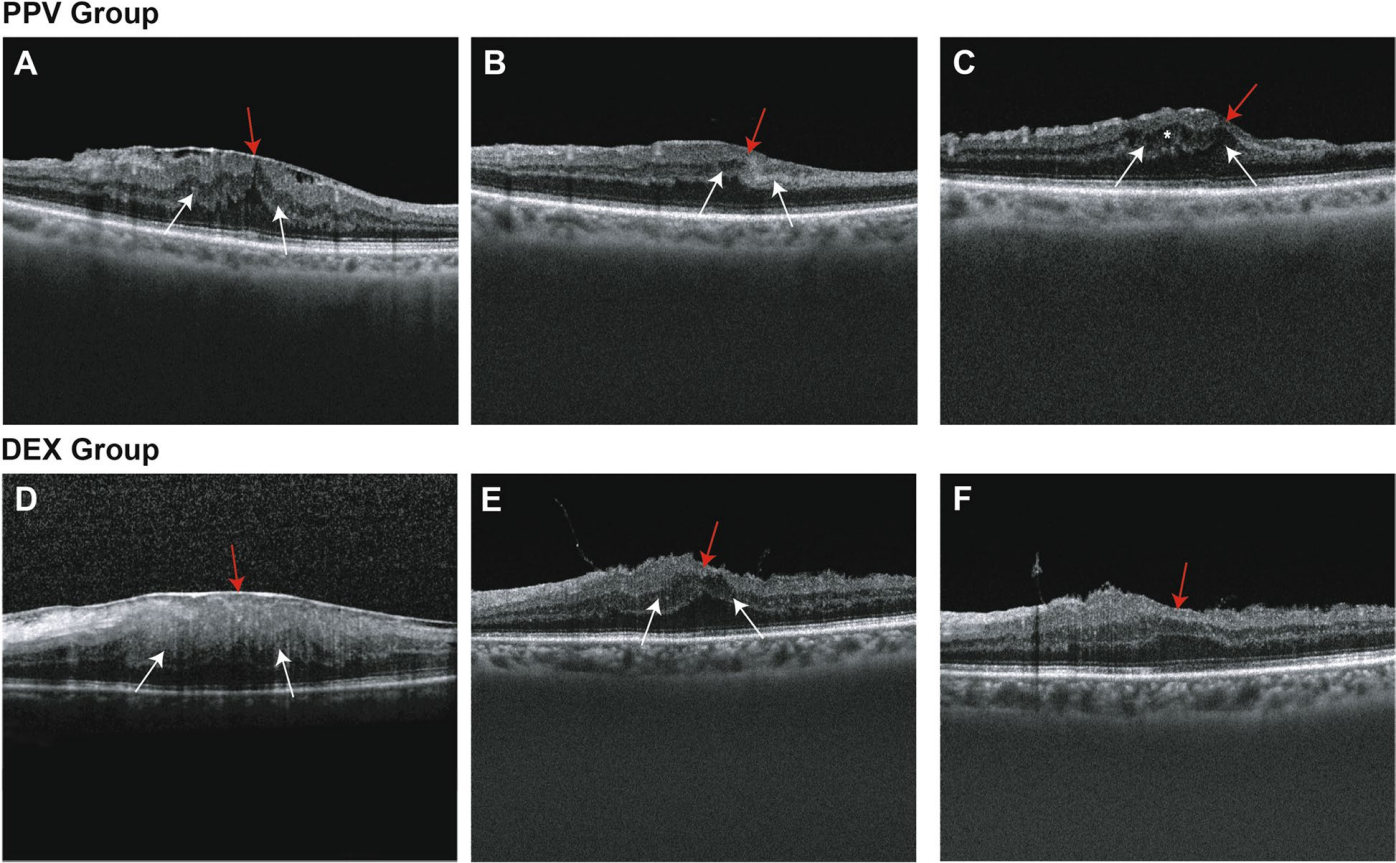
Preoperative and postoperative OCT images of patients in the PPV and DEX groups. **A** Preoperative OCT image in the PPV group with the presence of EIFL (red arrow) and mild DRIL (white arrow); **B** 1 month postoperatively with reduced CMT, presence of EIFL, and mild DRIL in the PPV group; **C** 6 months postoperatively with the presence of EIFL, mild DRIL and IRC (*) in the PPV group. **D** Preoperative OCT image in the DEX group with the presence of EIFL and severe DRIL; **E** 1 month postoperatively with reduced CMT, presence of EIFL, and mild DRIL in the DEX group; **F** 6 months postoperatively with the presence of EIFL and absent DRIL in the DEX group. EIFL (red arrow), DRIL (white arrow), IRC (*)

Severe IRC was not detected either preoperatively or postoperatively. Six months postoperatively, IRC was still present in 12 patients (60%) in the PPV group and 6 (28.6%) in the DEX group (*P* = 0.043). The IRC grade was significantly improved in the DEX group compared to that in the PPV group at 6 months, with a statistically significant difference (*P* = 0.038; Table 3). Figure 1 displays a series of anatomical changes in the macula region examined by OCT in the PPV and DEX groups.

**Table 3:** Comparison of 6-month OCT biomarkers between the two groups

	**PPV, *n* = 20**	**DEX, *n* = 21**	***T/Z***	***P***
**CMT(μm)**	**383.00 ± 84.78**	**375.38 ± 77.85**	**0.30**	**0.766**
**ΔCMT**	**78.40 ± 72.74**	**138.10 ± 97.60**	**2.21**	0.033^*^
**DRIL(absent/mild/severe)**	**9/10/1**	**16/5/0**	**2.08**	0.037^*^
**SRF(yes/no)**	**1/19**	**0/21**	**-**	**0.488**
**IRC(absent/mild/modrent/severe)**	**8/11/1/0**	**15/6/0/0**	**4.30**	0.038^*^
**IS-OS disruption(yes/no)**	**7/13**	**2/19**	**-**	**0.067**

^*^P < 0.05

### Dexamethasone implantation is more likely to produce an improvement in OCT biomarkers

The DRIL and IRC grades were combined to simplify the analysis, and Spearman correlation analysis was conducted to assess the baseline factors associated with the results for all patients. No association was found between sex, age, symptom duration, IERM stage, SRF, or postoperative outcomes. The intraoperative DEX implantation was associated with a significant CMT reduction (≥ 100 μm) (*r* = 0.463, *P* = 0.002), DRIL (*r* =- 0.320, *P* = 0.042), and IRC (*r* =- 0.317, *P* = 0.044) at 6 months postoperatively. A univariate logistic regression analysis of the clinical factors was performed based on the results of the correlation analysis to select variables that were predictive of postoperative outcomes. At 6 months, a significant reduction in CMT values (≥ 100 μm) from baseline values (odds ratio (OR), 7.47; 95% confidence intervals (CI), 1.87–29.88; *P* = 0.003), as well as improvements in DRIL (OR, 0.26; 95% CI, 0.07–0.97; *P* = 0.041) and IRC (OR, 0.27; 95% CI, 0.07–0.98; *P* = 0.043) were associated with the intraoperative implantation of DEX (Table 4).

**Table 4:**
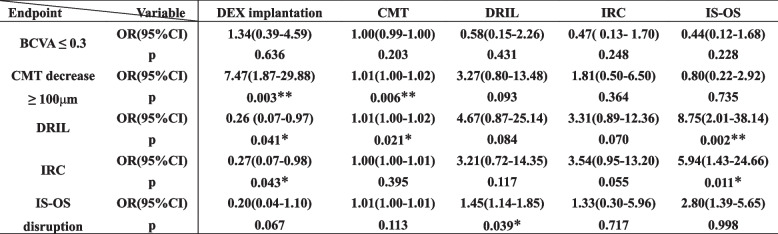
Univariate logistic analysis for predictors of 6-month outcomes in advanced IERM

^*^P < 0.05; ^**^P < 0.01

Multivariate logistic regression analyses were further conducted to determine predictors associated with 6-month outcomes. Given the limited sample size of this study, we only included variables with *P* < 0.05 in the univariate analysis for the multivariate analysis. Our results showed that patients with intraoperative DEX implantation were more likely to have a significant CMT reduction (≥ 100 μm) from baseline (OR, 9.44; 95% CI, 1.58–56.56; *P* = 0.014) and less likely to have DRIL at 6 months after surgery (OR, 0.08; 95% CI, 0.01–0.68; *P* = 0.021; Table 5).

**Table 5:** Multivariate logistic analysis for predictors of 6-month outcomes in advanced IERM

**Endpoint**	**Variable**	**OR(95%CI)**	***P***
**CMT decrease ≥ 100 μm**	**DEX implantation**	**9.44(1.58–56.56)**	0.014^*^
	**CMT**	**1.01(1.00–1.02)**	0.045^*^
	**DRIL**	**2.39(0.29–20.00)**	**0.421**
**DRIL**	**DEX implantation**	**0.08(0.01–0.68)**	0.021^*^
	**CMT**	**1.01(1.00–1.03)**	0.011^*^
	**IS-OS disruption**	**5.55(0.95–32.44)**	**0.057**
**IRC**	**DEX implantation**	**0.23(0.05–1.09)**	**0.065**
	**IRC**	**4.55(0.93–22.20)**	**0.061**
	**IS-OS disruption**	**4.55(0.97–21.38)**	**0.055**

^*^P < 0.05

## Discussion

Several studies have investigated the characteristics of OCT in patients with ERM to identify the anatomical changes in the macular region associated with visual acuity. The objective of our study was to observe changes in OCT biomarkers in patients with advanced IERM treated with intravitreal DEX implantation and to evaluate its anatomical and functional effects. Our results suggest that PPV combined with intravitreal DEX implantation effectively reduces CMT and improves DRIL in patients with advanced IERM.

Previous studies have suggested that patients with advanced IERM tend to have poorer postoperative functional and anatomical outcomes. For example, studies have found that the occurrence of postoperative macular edema and microcysts in the inner nuclear layer is correlated with the stage of IERM and that EIFL, DRIL, macular edema, and microcysts all have a significant impact on visual acuity [6, 19]. Mavi et al. studied 112 eyes of 112 patients with vitrectomy combined with ERM and internal limiting membrane (ILM) peeling and discovered that patients in the advanced stages (stages 3 and 4) had less visual and anatomical improvement [7]. In another study, Karasu et al. analyzed 138 eyes of 106 patients and found that EIFL severity was negatively associated with postoperative anatomical and visual acuity recovery [8]. Similarly, Mahm et al. analyzed 322 eyes with ERM and found that stage 4 ERM is associated with poor postoperative visual acuity [9].

DEX is a potent anti-inflammatory corticosteroid with pharmacokinetics indicating slow release in the vitreous cavity for up to 6 months [20]. DEX pharmacokinetics and release profiles were similar in patients treated with and without PPV [21]. Its safety and efficacy in patients undergoing vitrectomy have been demonstrated [22]. Iglicki et al. find similar anatomical and functional effects of DEX implants in non-vitrectomized and vitrectomized eyes [23].

However, the use of DEX in IERM remains unclear. Guidi et al. and Sane et al. found no significant changes in DEX implantation compared to PPV at 6 months of postoperative follow-up [24, 25]. Similarly, Savastano et al. found no significant benefit of intraoperative DEX implantation for ERM at long-term follow-up [26]. However, Chang et al. and Furino et al. applied DEX to treat refractory macular edema after ERM peeling, and the patient’s visual acuity improved significantly and macular edema regressed within 6 months after surgery [27, 28]. In another study, Iovino et al. applied DEX combined with PPV to treat stage 3–4 IERM with intraretinal cysts. The results showed that at 6 months after surgery, the BCVA in the DEX group was better than that in the control group, and the CMT was significantly lower than that in the control group. This was a clinical study involving fine grading of the preoperative macular structure [16].

No increase in IOP was observed in the DEX group during the follow-up. Our study demonstrated a significant increase in postoperative BCVA in both the DEX and PPV groups. However, there was no significant between-group difference in BCVA at different time points. Nevertheless, the DEX group showed significantly greater improvement in BCVA at 1 and 6 months. Additionally, the postoperative CMT was significantly lower than the preoperative value in both the groups. However, there was no significant difference in the CMT between the two groups at different time points.

Nonetheless, the DEX group exhibited a significantly greater reduction in CMT 1 and 6 months after surgery. Although there were no significant differences in the baseline characteristics, preoperative BCVA and CMT were worse in the DEX group than in the PPV group. Given the retrospective nature of this study, intravitreal DEX implantation may be considered a favorable option for patients with severe edema. In addition, these findings also suggest the effectiveness of DEX in improving anatomical and functional outcomes in patients with advanced IERM.

Upon analyzing the pre- and postoperative OCT characteristics, there were no statistically significant differences in the SRF and integration of IS-OS between the two groups at different time points. While no significant differences in the grades of DRIL and IRC were observed between the two groups preoperatively and at 1 month postoperatively, the results at 6 months postoperatively indicated a trend towards reduced DRIL and IRC with the use of DEX, suggesting a potential beneficial effect of DEX treatment in addressing the underlying pathological changes associated with IERM. In addition, multifactorial logistic regression analysis found that patients with intraoperative DEX implantation were more likely to have a significant CMT reduction (≥ 100 μm) from baseline and were less likely to have DRIL at 6 months postoperatively. DEX implantation is a predictor of postoperative anatomical improvement in patients with advanced IERM.

DRIL is defined as a condition where in the boundary between the ganglion cell-inner plexiform complex, inner nuclear layer, and outer plexiform layer cannot be identified [29]. DRIL is a common response to retinal stress in various retinal diseases; however, the pathogenesis of DRIL and its impact on BCVA remain unclear. Ishibashi et al. suggested that DRIL may be caused by damage to the retinal microcirculation, resulting in the destruction of neural structures and mechanical traction of the macula [30]. Sun et al. suggested that DRIL might disrupt the pathway that transmits visual information from photoreceptors to ganglion cells [31]. Zur et al. conducted a study to explore the impact of DRIL on the visual and structural prognosis in patients with IERM undergoing vitrectomy and ERM peeling. They found that the baseline BCVA, central foveal subfield thickness and maximal retinal thickness (MRT) were associated with DRIL severity. Patients with severe DRIL had limited surgical benefits compared to those with mild or no DRIL [13]. Karasavvidou et al. also investigated the prognostic impact of DRIL in patients with IERM and found that MRT and severe DRIL were strongly associated with worsening visual acuity in multivariate analysis [12]. As CMT decreased, the traction on the inner retinal layer decreased, which alleviated DRIL to some extent. Zur et al. studied DEX implantation for the treatment of diabetic macular edema and found that DEX implantation ameliorate DRIL [32].

The incidence of macular edema following IERM surgery can range from 13 to 64% [33, 34]. Severe cases of IERM with significant anatomical disruption are more prone to postoperative edema and have longer-lasting effects, impeding the rapid recovery of macular anatomy and visual function. Iuliano et al. reported a 14.3% occurrence of postoperative macular edema in patients with lower (1–3) stages of IERM and a 57.1% occurrence of postoperative macular edema in patients with stage 4 IERM [6]. A study involving 69 eyes with IERM found that preoperative intraretinal cystoid changes were the only risk factor for the development of intraretinal cystoids 3 months postoperatively [35]. Frisina et al. found that the presence of preoperative intraretinal cysts was related to persistent postoperative cystoid macular edema (CME) and that 10% of eyes without preoperative CME developed CME postoperatively [34].

Several studies have suggested that degenerating Müller cells may promote the formation of intraretinal cysts; ERM and ILM peeling may lead to the detachment and destruction of adherent Müller cell endfeet, resulting in the persistence and increase in CME [36, 37]. Vitreous traction is also a determinant of macular edema, leading to retinal distortion or disruption of the blood-retinal barrier [38]. Underlying inflammatory responses play a key role in this process. The postoperative release of various inflammatory factors and cytokines exacerbates blood-water barrier disruption, leading to fluid accumulation and persistent postoperative macular edema [17, 33]. To address this, vitreous cavity injection of corticosteroids can be considered an option to hasten the regression of postoperative edema. Corticosteroids have been shown to block the production of vascular endothelial growth factors and other inflammatory mediators, inhibit inflammatory responses, enhance vascular endothelial cell barrier function, and reduce macular edema [39, 40].

Our study highlights the potential benefits of DEX implantation as an adjunctive treatment in managing advanced IERM and suggests its potential to improve postoperative outcomes. This approach promises to refine the current standard of care and offer patients a more effective and comprehensive therapeutic option. Specifically, the DEX group demonstrated greater improvement in postoperative visual acuity and a more significant reduction in the CMT than the PPV group. Moreover, the severity of DRIL and IRC was significantly reduced at the 6-month follow-up in the DEX group compared to that in the PPV group. Importantly, we also found that intraoperative DEX implantation can serve as a useful predictor of significant CMT reduction (≥ 100 μm) and the absence of DRIL in patients with advanced IERM. However, larger sample sizes and longer follow-up periods are needed to confirm our findings.

Our study had some limitations. First, due to the retrospective nature of our study and the small sample size, the scientific evidence supporting our findings may be considered insufficient. Second, the follow-up period of 6 months may not have fully elucidated the long-term effects of intravitreal DEX implantation in patients with advanced IERM. Future research should consider assessing the impact of DEX implantation on macular anatomy and its correlation with postoperative visual outcomes.

## Conclusions

These findings suggest that combined vitrectomy and intravitreal DEX implantation may be a viable option for managing advanced IERM, particularly in patients with severe preoperative macular edema or severe DRIL. Vitrectomy combined with intravitreal DEX implantation facilitates the recovery of postoperative visual acuity and the improvement of anatomical outcomes in patients with advanced IERM, effectively reducing CMT and improving DRIL.

## Data Availability

The data supporting the findings of this study are available from the corresponding author upon reasonable request.

## Abbreviations

IERM: Idiopathic epiretinal membrane
DEX: Dexamethasone
PPV: Pars plana vitrectomy
BCVA: Best corrected visual acuity
CMT: Central macular thickness
DRIL: Disorganization of the retinal inner layers
SRF: Subretinal fluid
IRC: Intraretinal cystoid
IS-OS: Inner-outer segment layer
IOP: Intraocular pressure
SD-OCT: Spectral domain optical coherence tomography
EIFL: Ectopic internal foveal layer
IOL: Intraocular lens
logMAR: Logarithm of the minimal angle of resolution
ILM: Internal limiting membrane
MRT: Maximal retinal thickness
CME: Cystoid acular edema
